# The hospital policy on breastfeeding project to promote breastfeeding in Italy: not so ambitious, still effective

**DOI:** 10.1186/s13052-026-02249-y

**Published:** 2026-05-13

**Authors:** Riccardo Davanzo, Guglielmo Salvatori, Maria Elisabetta Baldassarre, Luigi Gagliardi, Elsa Viora, Elena Scarpato, Massimo Agosti

**Affiliations:** 1 Nutrition Research Centre, University of Insubria, Varese, Italy; 2 Neonatal Intensive Care Unit, Bambino Gesù Children’s Hospital, IRCCS, Rome, Italy; 3 Department of Interdisciplinary Medicine-Neonatology and NICU, University “Aldo Moro”, Bari, Italy; 4 Department of Mother and Child Health, AUSL Toscana Nord Ovest, Pisa, Italy; 5 President Elect, Italian Society of Obstetrics and Gynecology (SIGO), Turin, Italy; 6 Department of Translational Medical Sciences – Section of Pediatrics, University “Federico II”, Naples, Italy; 7 Pediatric Department, Hospital ‘F. Del Ponte’, University of Insubria, Varese, Italy

**Keywords:** Breastfeeding, Skin-to-skin contact, Rooming-in, Training, Hospital policy, Baby Friendly Hospital Initiative

## Abstract

**Background:**

According to the available data, the breastfeeding rate at hospital discharge is suboptimal in Italy. The present study aims to evaluate the effectiveness of the Hospital Policy on Breastfeeding (HPB) Project launched by the Italian scientific societies specialising in perinatal care to promote breastfeeding in maternity hospitals (MHs).

**Methods:**

The HPB project was designed as an open before-after study to increase the rate of exclusive breastfeeding at hospital discharge in a population of healthy term infants with a normal birth weight following a bundle intervention. The primary outcome was the breastfeeding rate at hospital discharge. The secondary outcomes were data on the elements of the bundle intervention: 1) establishment of a local hospital breastfeeding working group, 2) adoption of a breastfeeding policy, 3) breastfeeding training for perinatal care professionals, 4) enhanced implementation of skin-to-skin contact practice at childbirth and mother-baby rooming-in, and 5) development and/or improvement of breastfeeding-related protocols.

**Results:**

Of the 104 hospitals that were initially enrolled in a two-years study between February 2023 and January 2025, 97 completed it. The exclusive breastfeeding rate and the fully breastfeeding rate of healthy term newborns with a normal birth weight at hospital discharge increased from 67.3% to 71.0% (*p* < 0.001), and from 69.3% to 71.3% (*p* < 0.001), respectively over a four-month period. Moreover, all secondary outcomes improved. Specifically: 1) more MHs set up a multidisciplinary breastfeeding working group, developed breastfeeding policy and prepared the breastfeeding-related protocols, 2) the proportion of perinatal healthcare workers trained in breastfeeding increased from 55.9% up to 71.7% (*p* < 0.001), and 3) the proportion of newborns who experienced skin-to-skin contact in the delivery room after vaginal delivery increased from 76.9% to 89.4% (*p* < 0.001), as did the proportion of newborns who experienced rooming-in, from 83.9% to 85.4% (*p* < 0.05), as measured over a one-month period.

**Conclusion:**

The HPB Project successfully promoted exclusive breastfeeding at hospital discharge, as well as skin-to-skin contact and rooming-in practices, among healthy term infants with a normal birth weight, in a large sample of Italian MHs.

**Supplementary Information:**

The online version contains supplementary material available at 10.1186/s13052-026-02249-y

## Background

The benefits of breastfeeding and mother’s milk are well known, not only for the mother-infant dyad, but also for the family as a whole [1]]. Current national and international infant feeding recommendations support exclusive breastfeeding for the first six months of life, followed by continued breastfeeding alongside complementary foods and occasionally up to the second year of life and beyond [2–6].

Searching PubMed for the term ‘breastfeeding’ yields over 76,500 references (as of January 2026). While this extensive body of literature provides comprehensive information on facilitating breastfeeding in healthcare services, it is important to recognize that this is often done incompletely or imperfectly [7]. A lack of professional interest in breastfeeding and/or conflicts of interest of various magnitudes are often cited as key causes [8]. Over the past 50 years, women, scientific societies and health agencies, have increasingly emphasised the importance of breastfeeding and urged health professionals to proactively promote it in a competent and unambiguous manner [9–13].

In 1991, UNICEF launched the commendable Baby Friendly Hospital Initiative (BFHI), based on a clear vision of how to transform the culture and organization of maternity hospitals (MHs) to promote, protect and support breastfeeding. In the subsequent decades, hospitals adhering to the BFHI have effectively achieved this goal, proving more resilient in challenging situations such as the pandemic caused by the SARS-CoV-2 virus [14]. Unfortunately, since the international launch of the BFHI, only around thirty out of a total of 354 MHs in Italy have been accredited as Baby Friendly [15]. A non-exhaustive list of potential reasons is provided below.

In Italy there are no significant government incentives for hospitals to become Baby Friendly. Although some Italian regions and autonomous provinces have chosen to promote the BFHI among their health facilities, strict, specific work objectives for the directors of health authorities have rarely been set.

Despite producing a series of significant documents on breastfeeding between 2012 and 2024, the Technical Task Force on Breastfeeding (TAS) of the Ministry of Health [16] has been given neither sufficient autonomy of action nor a dedicated budget. This would enable more effective interventions to promote breastfeeding, particularly the BFHI. Furthermore, the three-year mandate of the TAS expired in November 2024, without being reconfirmed.

The UNICEF organisation to support hospitals in achieving the BFHI goal includes tutoring and external evaluation to certify hospitals’ achievements [17]. Becoming a BFH can be challenging [18], particularly in Italy today, given the shortage of healthcare staff, high staff turnover and the fact that the local BFHI implementation still relies heavily on the positive attitude of healthcare workers and capable, enthusiastic team leaders. Furthermore, once achieved, BFH status, is difficult to sustain [19], leading to the need for demanding periodic re-certifications.

Although there is no national monitoring of breastfeeding in Italy, available data show low breastfeeding rates [20] particularly at hospital discharge [21]. In fact, a 2014 Survey conducted by the TAS found that the rate of exclusive breastfeeding rate at discharge from Italian MHs ranged widely from 20% to 97%, without providing an overall national figure [22]. Comparisons with other industrialized countries are not straightforward, as data recording may lack standardised methodology [23]. The exclusive breastfeeding initiation rates are 81% in the UK [24], 72% in Sweden [25] and 61.2% and 55.6% in BFH-accredited and non-accredited French MHs, respectively [26]. Furhermore, a study conducted in 11 European Countries found that the rate of any breastfeeding at birth varies between 56% and 98% [27], without specifying the percentage of neonates who were given infant formula during their hospital stay.

Against this background, and acknowledging the need for MHs to support the initiation of breastfeeding, the Breastfeeding Section of the Italian Society of Neonatology (COM.A.SIN) launched the Hospital Policy on Breastfeeding (HPB) Project in early 2023 as a joint initiative of the Italian scientific societies involved in perinatal care, including the Italian Society of Neonatology (SIN), the Italian Society of Pediatrics (SIP), the Italian Society of Pediatric Nutrition (SINUPE), the Italian Society of Obstetricians and Gynecologists (SIGO), the Italian Association of Hospital Obstetricians & Gynecologists (AOGOI), the Italian Society of Neonatal Nurses (SININF) and the Italian Society of Pediatric Nurses (SIPINF), together with the National Midwife (FNOPO) and Nurse (FNOPI) Boards and with Vivere Onlus, a family association.

The present study tested the hypothesis that the HPB Project, previously described elsewhere [28], could ultimately increase the exclusive breastfeeding rate at hospital discharge in a sample of Italian MHs through a series of interventions on breastfeeding policies and protocols, staff training, and postnatal practices.

## Methods

Between February and May 2023, 104 Italian MHs were enrolled in the HPB Project [28]. Italy has a population of 58.9 million, with just around 370,000 babies born in 2024. The mean maternal age at childbirth in Italy has gradually increased, and it is now 32.6 years of age [29].

In brief, the HPB Project is a 2-year open before-after study designed to assess the efficacy of a bundle intervention in increasing the exclusive breastfeeding rate of healthy term (≥37 weeks gestational age) newborns, with normal birth weight (≥2500 g).

The components of the bundle intervention designed to promote breastfeeding are as follows:

The establishment of a local hospital breastfeeding working group (BWG), including at least a pediatrician/neonatologist, a nurse, an obstetrician/gynecologist and a midwife, and possibly an anaesthetist and a family representative.

Adoption of a hospital policy on breastfeeding, that includes avoiding the prescription of formula milk at hospital discharge for breastfed newborn infants, whenever breastfeeding mothers are self-effective.

Activation and/or reinforcement of an on-site and/or online training programme on breastfeeding for perinatal healthcare workers, in accordance with the Ministry of Health’s guidance [30]. SIN, SIP, SIPINF, SININF, AOGOI, SIGO and FNOPO have prepared a series of structured courses on breastfeeding.

Development of 20 protocols covering various issues related to breastfeeding.

Implementation and/or reinforcement of the two main postnatal practices known to facilitate breastfeeding: skin-to-skin contact (SSC) at birth [31], and mother-neonate rooming-in [32, 33].

Monitoring of breastfeeding rates at hospital discharge using the 1991 WHO definitions on infant feeding [34].

The breastfeeding rate (the primary outcome) was calculated over two periods: between 1 June and 30 September 2023 (T1), and between 1 October 2024 and 31 January 2025 (T2). The secondary outcomes included: 1) implementation of a BWG; 2) development of hospital policies and protocols on breastfeeding, 3) implementation and/or reinforcement of SSC at childbirth and mother-baby rooming-in, and 4) completion of a breastfeeding training program for healthcare workers in the perinatal area.

Skin-to-skin contact was defined as holding the naked baby against the mother’s skin during the first 2 hours after birth [31]. Rooming-in [33] was defined as the mother and baby sharing a room for at least 20/24 hours (extensive rooming-in), with a maximum separation of four-hours between mother and baby, including visits, procedures or the mother voluntarily entrusting of the newborn to nursery staff.

The effective implementation of 20/24-hours rooming-in, from childbirth until the end of the hospital stay, was assessed using a questionnaire given to mothers when they were discharged from hospital. Data on SSC and rooming-in were collected ove a one-month period: from 15 September to 15 October 2023 (T1) and from 15 November to 15 December 2024 (T2).

The participating MHs reported data at T1 and T2 through SurveyMonkey platform in December 2023 and February 2025 (Supplementary Materials).

Statistical analysis. Descriptive data are reported as number of observations or percentages. The chi-square test was used to compare the breastfeeding rate between the pre-intervention (T1) and post-intervention (T2) groups. Comparison of changes in hospitals’ procedures at T1 and T2 were made using the McNemar test (a paired sample). A *p*-value less than 0.05 was considered statistically significant.

The sample size was calculated to detect a minimum absolute increase of 2% in the primary outcome (the rate breastfeeding rate), assuming a baseline prevalence of 50%. With a two-sided significance level (α) of 0.05 and a statistical power of 90%, approximately 13,100 newborns were required per group. However, this sample size was much lower than the estimated number of healthy newborns infants born over a four-month period in the 97 MHs enrolled in the study. Consequently, data on the breastfeeding rate at hospital discharge were limited to a four-month period.

## Results

### Characteristics of the study sample

The HPB Project enrolled 104 MHs, that account for around 29.4% of the 354 Italian MHs registered in 2022 [35] and an estimated 32% of all annual births in Italy. During the first year of the HPB Project, two MHs were closed before T1, and other five withdrew from the Project before T2. Thus, the present study’s data refer to the 97 MHs that completed both the T1 and the T2 online surveys (93.3% of the initial sample). This heterogeneous group of MHs is located in 13 out of 21 Italian regions and autonomous provinces. It consists of smaller local hospitals as well as university and tertiary care centres, thus providing different levels of care for childbirths ranging from fewer than 500 to more than 3000 per year, per single center. The main features of these 97 MHs are shown in Table 1.

**Table 1:** Main characteristics of the 97 Italian MHs that completed the intervention study

		MHs (N)	Percentage
Geographical distribution	Northern Italy	74	76.3%
	Central Italy	12	12.4%
	Southern Italy	11	11.3%
Type of contract with the National Health System	Public MH	96	99%
	Private MH	1	1%
Relationship with BFHI	None	84	86.6%
	Certified Baby Friendly Hospital	7	7.2%
	MH devoted to become Baby Friendly	6	6.2%
University Hospital	Yes	23	23.7%
	No	74	76.3%
MHs with a NICU	Yes	37	38.1%
	No	60	61.9%
Life births per year (2023)	<500	14	14.4%
	501–1000	38	39.2%
	1001–1500	17	17.5%
	1501–2000	12	12.4%
	2001–2500	6	6.2%
	2501–3000	7	7.2%
	>3000	3	3.1%
Cesarean section rate (2023)	≤20%	21	21.6%
	21–30%	56	57.7%
	31–40%	12	12.4%
	40–50%	6	6.2%
	51–60%	2	2.1%

### Type of feeding at hospital discharge

Following implementation of the HPB Project, the rate of exclusive breastfeeding at hospital discharge among healthy, normal weight, full-term infants significantly increased from 67.3% to 71.0% (*p* < 0.001) (Table 2). Despite the concurrent reduction in predominant breastfeeding rate (2.0% vs 0.3%), the difference in the fully breastfeeding rate between T1 (69.3%) and T2 (71.3%) remained significantly higher (*p* < 0.001).

**Table 2:** Breastfeeding rate at hospital discharge among healthy term neonates of 97 MHs

	T1 (November 2023)		T2 (February 2025)		Statistical analysis
Type of feeding at hospital discharge of healthy term newborn infants with BW ≥ 2500 g #	Number	Percentage	Number	Percentage	
a) Exclusive breastfeeding	24.046	67.3%	23.540	71.0%	p < 0.001
b) Predominant breastfeeding	720	2.0%	106	0.3%	-
c) Fully breastfeeding (a+b)	24.766	69.3%	23.646	71.3%	p < 0.001
d) Complementary feeding	8.720	24.4%	7.878	23.7%	-
e) No breastfeeding (formula only)	1.553	4.3%	1.643	5.0%	-
Total discharged neonates with feeding categorization	35.039	100%	33.167	100%	-

# Data recorded during 4 months in the same 97 MHs both at T1 and at T2

### Breastfeeding policy and working group

Following the bundle intervention, a breastfeeding policy was implemented in 91.7% of centres (Table 3). The contents of the breastfeeding policy were revised according the checklist in Table 4.

**Table 3:** Increased availability of the breastfeeding working group and the breastfeeding policy, and implementation of SSC and rooming-in in 97 MHs during the HPB Project

	T1 (November 2023)		T2 (February 2025)		Statistical analysis
	MHs fulfilling the requirement (Number/97)	Percentage	MHs fulfilling the requirement (Number/97)	Percentage	
Hospital Breastfeeding Working Group •Multi-professional •Anesthetist inclusive •Family representative inclusive	94 62 35	96.9% 63.9% 36.0%	97 72 58	100% 74.2% 59.8%	p = 0.07 p = 0.12 p < 0.01
Policy on breastfeeding available	42	43.3%	89	91.7%	p < 0.001*
Practice of SSC implemented •Only after vaginal delivery •After vaginal delivery as well as cesarean section •SSC not practiced	45 47 5	46.4% 48.4% 5.2%	36 60 1	37.1% 61.9% 1.0%	p = 0.07
Practice of rooming-in (≥20/24 hours) between mother and neonate implemented •Yes •No	88 9	90.7% 9.3%	94 3	96.9% 3.1%	p = 0.03 *

*McNemar’s test

MH: Maternity Hospital; SSC: skin-to-skin contact

**Table 4:** Topics to be covered by the policy on breastfeeding of the participating MHs

1.Approval by Hospital/Health Authority director
2. Communication to all staff
3. Communication to mothers/families (via website and/or poster and/or brochure)
4. Institution of a Maternity Hospital/Health Authority BWG
5. Overt statement that hospital staff is committed to promote and support breastfeeding
6. Revision of the contents of the antenatal classes in order to give pregnant women appropriate information on breastfeeding
7. Respect of the informed choice of a mother not to breastfeed
8. Implementation of SSC, rooming-in and responsive breastfeeding
9. Involvement of the Breastfeeding Working Group in case of future changes of postnatal practices possibly affecting breastfeeding
10. Information to mothers on the available community resources to support breastfeeding
11. Information to mothers on the availability of volunteer consultants to support breastfeeding in the community (e.g La Leche League)
12. Avoidance of formula milk prescription to self-effective breastfeeding mothers
13. Need for training on breastfeeding of the hospital staff
14. Breastfeeding monitoring at hospital discharge

By T2, all MHs reported having a multi-professional BWG, although only 59.8% included a family representative.

### Postnatal practices that facilitate breastfeeding

a) *Skin-to skin contact*. The SSC after vaginal delivery increased from 94.8% to 99.0% of MHs (Table 3). It was experienced also by a higher percentage of newborn infants eligible for this practice (76.9% vs 89.4%)(*p* < 0.001)(Table 5). The duration of the SSC during the first two hours of life was only recorded at T2, following the national implementation of a checklist for the prevention of postnatal collapse by the SIN in 2023 [36, 37]. At T2 the duration of SSC in the first two hours of life was appropriately longer than 60 minutes in 4.640 out of 5.325 (87.1%) healthy term neonates.

**Table 5:** Skin-to-skin contact and rooming-in among healthy term neonates in 97 MHs

	T1 (November 2023)		T2 (February 2025)		Statistical analysis
	Neonates who practiced/candidate neonates (n/N)	Percentage	Neonates who practiced/candidate neonates +(n/N)	Percentage	
Healthy term neonates who experienced SSC in the delivery room after vaginal delivery §	5.066/6.587	76.9%	5.412/6.054	89.4%	p < 0.001
Healthy term neonates with BW ≥ 2500 g who experienced extensive rooming-in (at least 20/24 hours) #	5.823/6.940	83.9%	6.435/7533	85.4%	p = 0.011

§ Data recorded during 1 month in 88 MHs at T1 and in 93 MHs at T2

# Data recorded from questionnaires submitted to mothers at hospital discharge during 1 month in 88 MHs at T1 and in 94 MHs at T2

SSC: skin-to-skin contact

b) *Rooming-in*. The proportion of MHs practicing an extensive rooming-in (at least 20/24 hours) increased from 90.7% to 96.9% (Table 3), while the proportion of healthy neonates with a birth weight > 2500 g, who experienced this practice, increased from 83.9% to 85.4% (*p*  =0.011) (Table 5).

### Breastfeeding training

The percentage of healthcare workers involved in perinatal care and trained in breastfeeding increased significantly from 55.9% to 71.7% (Table 6) across all care sectors. Healthcare assistants maintain lower coverage when compared to physicians, midwives and nurses.

**Table 6:** Training on breastfeeding of healthcare workers in 97 Italian MHs participating to the HPB Project

	T1 (November 2023)		T2 (February 2025)		
Healthcare workers per site of work	Trained/total health workers (n/N)	Percentage	Trained/total health workers (n/N)	Percentage	Statistical analysis
*Obstetrics and Gynecology Department*					
•Obstetricians/Gynecologists	435/1.384	31.4%	729/1.409	51.7%	
•Midwives	2.177/3.170	68.7%	2.882/3.361	85.7%	
•Nurses	269/630	42.7%	289/518	55.8%	
•Health care assistants	237/970	24.4%	413/1.012	40.8%	
Subtotal	3.118/6.154	50.7%	4.313/6.300	68.5%	
*Pediatric Department/Nursery*					
•Pediatricians	276/476	58.0%	229/336	68.2%	
•Nurses	663/952	69.6%	652/846	77.1%	
•Health care assistants	53/197	26.9%	71/175	40.6%	
Subtotal	992/1.625	61.0%	952/1.357	70.1%	
*Neonatology Department/Nursery*					
•Neonatologists	447/662	67.5%	553/682	81.7%	
•Nurses	1.303/1.966	66.3%	1.626/1.977	82.2%	
•Health care assistants	110/281	39.1%	176/308	57.1%	
Subtotal	1.860/2.909	63.9%	2.355/2.967	79.3%	
Total (all perinatal staff)	5.970/10.688	55.9%	7.620/10.624	71.7%	p < 0.001

### Breastfeeding protocols

At the start of the HPB Project, MHs had only partially implemented protocols related to breastfeeding, with the exception of the protocol on neonatal hypoglycemia. By T2, however, each protocol was available in at least 78% of the centres (Table 7).

**Table 7:** Protocols related to breastfeeding in 97 MHs. At T1 protocols are listed in decreasing order of availability. Every protocol is highlighted in a color according to the percentage of its implementation in the 97 MHs: grey for <50%, yellow for 51–70%, green color for >70%

	T1 (November 2023)		T2 (February 2025)		
	Hospitals that adopted the protocol (N)	Percentage	Hospitals that adopted the protocol (N)	Percentage	p value *
1. Prevention of neonatal hypoglycemia	91	93.8%	91	93.8%	-
2. SSC after vaginal delivery	85	88.5%	95	97.9%	0.002
3. Check list on breastfeeding topics for antenatal classes	77	79.4%	87	89.7%	0.002
4. Prevention of SUPC	73	75.2%	88	90.7%	0.001
5. Storage of expressed mother’s milk and human donor milk	70	72.2%	92	94.8%	<0.001
6. Zero separation and rooming-in 24/24 h	69	71.3%	91	93.8%	<0.001
7. Hospital discharge of the breastfed newborn	60	61.8%	88	90.7%	<0.001
8. Prevention of in hospital neonatal fall	59	60.8%	85	87.6%	<0.001
9. Responsive exclusive breastfeeding	59	60.8%	84	86.6%	<0.001
10. Helping mothers to breastfeed	58	59.8%	87	89.7%	<0.001
11. Expression of breast milk	54	55.7%	76	78.3%	<0.001
12. Prevention and management of pain while breastfeeding	54	55.7%	86	88.7%	<0.001
13. SSC after CS	52	53.6%	86	88.7%	<0.001
14. Supplementing breast milk with formula	52	53.6%	83	85.6%	<0.001
15. Contraindications to breastfeeding	51	53.6%	81	83.5%	<0.001
16. Thermal control of the neonate	50	51.5%	86	88.7%	<0.0001
17. Prevention and management of breast engorgement	50	51.5%	88	90.7%	<0.001
18. Prevention and management of lactational mastitis	49	50.5%	85	87.6%	<0.001
19. Management of early neonatal weight loss	48	49.5%	78	80.4%	<0.001
20. How to support breastfeeding in the jaundiced newborn infant during phototherapy	46	47.4%	83	85.6%	<0.001

* McNemar’s test

SSC = skin-to-skin contact; SUPC: sudden unexpected postnatal collapse; CS: caesarean section

## Discussion

Although inspired by the BFHI, the HPB Project requires hospitals to achieve less ambitious goals, including those set out in the International Code of Marketing of Breast-milk Substitutes (“the Code”) [38] in order to facilitate greater adherence, in the short time. The HPB Project intends to encourage many MHs to promote breastfeeding to some extent, rather than aiming to achieve the gold standard in just a few of them. The HPB Project was attractive to MHs because there was no direct local financial burden related to subscription. Furthermore, the collaborative atmosphere and the provision of distance learning courses and breastfeeding protocols were well received.

The quality of the data provided by the MHs was systematically verified online in agreement with the local referents. Unlike the BFHI, the HPB Project does not carry out site visits or provide external evaluations of breastfeeding achievements. Although external evaluation is more accurate than self-evaluation [39], the latter option made the project more feasible. Clearly, a MHs participating in the HPB Project initially may later follow the BFHI pathway, or vice versa as evidenced by the participation to the HPB Project of seven MHs already accredited by UNICEF.

The HPB Project’s effectiveness is demonstrated by the significant, albeit small, increase in exclusive breastfeeding rate at hospital discharge (the primary outcome), which reached 71.0% in a population of over 33.000 neonates, accounting for almost 8.9% births in Italy in 2024 [29].

Nevertheless, this figure is still not satisfactory in absolute terms, as it refers to a selected population of healthy, term neonates with a normal birth weight, for whom we would expect a better breastfeeding outcome at hospital discharge, with an exclusive breastfeeding rate of at least 80% [17]

All MHs report constituting of a BWG with increased family representative inclusion, who can provide a valuable first-hand insight into the emotional needs of breastfeeding parents and the importance of support systems. By incorporating the lived experiences of families, working groups can develop more realistic guidelines that will ultimately lead to better breastfeeding outcomes.

The vast majority of MHs also established a breastfeeding policy, that could set organizational and clinical standards for healthcare professionals [40–42].

Among the MHs participating in the study the practice of SSC increased significantly over time. SSC is usually recommended to be initiated immediately after birth, and continued it uninterruptedly for at least one hour, or until the first breastfeed [43]. Unfortunately, we have no information on how long the practice was carried out for at the beginning of the study, but we suspect it may have been for a shorter period of time. In 2023, the SIN had nationally implemented a checklist for the prevention of postnatal collapse [37]. As this new checklist includes a record of the duration of SSC in the first two hours of life, we can now confirm that the vast majority of healthy, term neonates experienced SSC for more than 60 minutes in the first two hours after birth, which is the minimum length commonly considered necessary to generate benefits [44].

According to the zero-separation policy, rooming-in for mother and baby during hospital stay after childbirth is widely recommended [17, 45, 46]. However, due to the possibility of maternal mental distress or the need for clinical procedures in the early postpartum period [47, 48], the HPB Project recognises that a baby may need to be cared for separately by healthcare personnel for up to 4 hours out of every 24 [28]. In our study, rooming-in has been successfully increased, albeit modestly, and is not yet routinely applied in all MHs.

Furthermore, the HPB Project significantly increased in-service training on breastfeeding among hospital healthcare professionals in the perinatal area [49–51]. These professionals have a duty to promote breastfeeding, regardless of their personal experience with infant feeding or their specific day-to-day activities [52, 53]. Despite the notable expansion in breastfeeding training for each health professional group (pediatricians/neonatologists, nurses, obstetricians/gynecologists and midwives), healthcare assistants remain disproportionately overlooked. This is particularly concerning given their important role in providing personalised care to breastfeeding mothers on a daily basis within hospital wards. Although healthcare assistants are not breastfeeding specialists, they can play a crucial role in providing families with the appropriate support.

The relatively rapid increase in training coverage observed in our sample of MHs over a two-year period was primarily achieved through the use of online breastfeeding training resources, which complemented traditional in-person training. This approach was useful in overcoming the constraint of the significant shortage of hospital staff in Italy, that hinders the healthcare workers’ ability to engage in on-site training initiatives.

Finally, the present study demonstrates the widespread dissemination of healthcare protocols, tailored to specific hospital practices among MHs, ensuring consistent, evidence-based care for breastfeeding mothers and infants [54].

This study has also some limitations. Firstly, the enrolled MHs represent a large but self-selecting group who were more motivated to promote breastfeeding, which might have contributed to the success of the intervention. Therefore, we cannot assume that the breastfeeding rate observed in this study is representative of all Italian hospitals. Secondly, the sample of MHs may be more representative of northern Italy, given that more facilities from this part of the country participated to the HPB Project. Thirdly, seven out of the 104 MHs initially enrolled in the HPB Project were lost to follow-up and excluded from the data analysis, accounting for 6.7% of the study sample. Fourthly, the HPB Project focuses on hospitals as they are critical for breastfeeding initiation. This ignores multiple personal, familial, cultural, and socio-economical influencing factors that act after hospital discharge and in the community. These factors are poorly influenced by hospital healthcare professionals [55]. In particular, we recognise that community healthcare professionals play a pivotal role in the overall success of breastfeeding, which requires a positive attitude, specific skills and continuity of care.

The present study was conducted independently of the BFHI, that is a widely recognized and well reputed international initiative to promote, protect and support breastfeeding in MHs [17]. Although the BFHI and the HPB Project share the same objective of promoting breastfeeding, the latter is characterised by a pragmatic approach with standards of care that are easier for MHs to achieve. Scientific societies, meanwhile, have the institutional position and the competence to interact positively and empathetically with hospital healthcare workers while conducting the HPB Project.

Unlike the BFHI, the HPB Project is less strict about the adherence to the Code, accepting open collaboration between healthcare professionals and the baby-food industry [56], but without encouraging behaviours that are detrimental to infant health, professional dignity or Italian law [57]. In the absence of legal obligations, each professional must therefore address the dilemma of the Code on a personal level. This approach aims to remove the issue of the mandatory adherence to the Code as a reason, or perhaps an excuse, for a lack of commitment to promoting breastfeeding among healthcare professionals. Finally, it must be recognised that the HPB Project still prohibits the prescription of milk formula for newborns successfully breastfed newborns and prohibits the presence of pre-printed spaces for the prescription of a milk formula in discharge reports.

## Conclusions

The present study demonstrates the effectiveness of a bundle intervention in promoting breastfeeding within a relatively short period of time, in a large group of MHs, that voluntarily and concurrently enrolled in a nationwide project run by healthcare professionals within the National Health System. These MHs were not ready or not yet ready to face the challenges of the BFHI.

Although the exclusive breastfeeding rate at hospital discharge for healthy term neonates has improved, it remains suboptimal and requires further efforts and continuous monitoring.

Cooperative interaction between the BFHI and the HPB Project would enhance breastfeeding promotion at a national level, provided that common ground is sought and appreciated rather than differences.

## Electronic supplementary material

Below is the link to the electronic supplementary material.

Supplementary material 1

## Data Availability

The datasets used during the current study are available from the corresponding author on reasonable request.

## Abbreviations

AOGOI: The Italian Association of Hospital Obstetricians and Gynecologists
BFHI: Baby Friendly Hospital Initiative
BWG: Breastfeeding Working Group
COM.A.SIN: Breastfeeding Section of the Italian Society of Neonatology
FNOPI: National Nurse Board
FNOPO: National Midwife Board
GA: Gestational age
NICU: Neonatal Intensive Care Unit
SIGO: Italian Society of Obstetricians and Gynecologists
SIN: Italian Society of Neonatology
SININF: Italian Society of Neonatal Nurses
SINUPE: Italian Society of Pediatric Nutrition
SIP: Italian Society of Pediatrics
SIPINF: Italian Society of Pediatric Nurses
SSC: Skin-to-Skin Contact
SUPC: Sudden Unexpected Postnatal Collapse
TAS: Task Force on Breastfeeding
TASIP: Breastfeeding Section of the Italian Society of Pediatrics
UNICEF: United Nations International Children’s Fund
WHO: World Health Organization
