# Supplementary material for: The hospital policy on breastfeeding project to promote breastfeeding in Italy: not so ambitious, still effective
Source: Ital J Pediatr. 2026 May 13;52:119. doi: 10.1186/s13052-026-02249-y (PMC13445820; doi:10.1186/s13052-026-02249-y)
Supplement: Supplementary file 1 [file 13052_2026_2249_MOESM1_ESM.docx]

***DATA COLLECTION FORM; HPB Project***

Each Maternity Hospital (MH) should provide its own form.

# **Part A. Information on the MH**

# 1. Heath Authority:

# 2. MH identification number

# 3. MH name:

# 4. Leader of the breastfeeding working group BWG (responsible of data transmission):

a) Name
b) Surname
c) Mobile number:

5. Is your MH a baby friendly hospital?

a) Yes

b) No

c) No; nevertheless, the MH in on the BFH pathway

| **Part B. BWG** | | |
| --- | --- | --- |
|  | Yes | No |
| a) Does a working group on breastfeeding (BWG) exist in your MH? |  |  |
| b) Is it truly multidisciplinary, including at least a pediatrician/neonatologist, an obstetrician, a midwife and a nurse? |  |  |
| c) Does it include an anesthesiologist? |  |  |
| d) Does it include a family representative? |  |  |

| **Part C. Hospital policy on breastfeeding** | **Yes** | **No** |
| --- | --- | --- |
| 1. Approval by Hospital/Health Authority director |  |  |
| 2. Communication to all staff |  |  |
| 3. Communication to mothers/families    a. via website    b. via poster    c. via brochure |  |  |
| 4. Institution of a Maternity Hospital/Health Authority breastfeeding working group |  |  |
| 5. Overt statement that hospital staff is committed to promote and support breastfeeding |  |  |
| 6. Revision of the contents of the antenatal classes in order to give pregnant women appropriate information on breastfeeding |  |  |
| 7. Respect of the informed choice of a mother not to breastfeed |  |  |
| 8. Implementation of SSC, rooming-in and responsive breastfeeding |  |  |
| 9. Involvement of the breastfeeding working group in case of future changes of postnatal practices possibly affecting breastfeeding |  |  |
| 10. Information to mothers on the available community resources to support breastfeeding |  |  |
| 11. Information to mothers on the availability of volunteer consultants to support breastfeeding in the community (e.g La Leche League) |  |  |
| 12. Avoidance of formula milk prescription to self-effective breastfeeding mothers |  |  |
| 13. Need for training on breastfeeding of the hospital staff |  |  |
| 14. The need for breastfeeding monitoring at hospital discharge |  |  |

| **Part D. Postnatal practices facilitating breastfeeding**  Tick the correct box | |
| --- | --- |
| Is SSC during at least 2 hours practiced with newborn infants **≥**37 GA? | |
| a) No |  |
| b) Yes, only after vaginal delivery |  |
| c) Yes, also after CS |  |
| Does the MH use check list by the Italian Society of Neonatology on the prevention of SUPC prevention? |  |
| a) Yes |  |
| b) Yes, although just beginning the implementation |  |
| c) No |  |
| Which is the number of SSC after vaginal delivery among healthy newborn infants **≥** 37 weeks GA during one-month period? |  |
| a) Number of SSC during one month |  |
| b) Total number of healthy newborn infants **≥** 37 weeks GA discharged during one month |  |
| Is extensive rooming-in (rooming-in >20 hours/24) practiced among healthy term newborn infants with a birth weight ≥ 2500 g? | |
| a) Yes |  |
| b) No |  |
| Which is the number of dyads who experience extensive rooming-in among healthy newborn infants **≥** 37 weeks GA with BW ≥ 2500 g in one-month period? |  |
| a) Number of extensive rooming-in during one-month period |  |
| b) Total number of healthy newborn infants **≥** 37 weeks GA with BW ≥ 2500 g discharged during one month |  |

| **Part E. Breastfeeding rate at hospital discharge** (according to Infant feeding definition by WHO, 1991). Data refer to healthy newborn infants **≥** 37 weeks GA and with a BW ≥ 2500 g during the period from 1 June until 30 September 2023. | |
| --- | --- |
|  | Number of neonates |
| - Mother’own milk |  |
| - Donor milk |  |
| - PBF |  |
| - Complementary feeding |  |
| - Formula feeding |  |
| - Total |  |

| **Part F. Training on breastfeeding of health workers** | |
| --- | --- |
| Health workers per site of work | Trained/total health workers  (N) |
| *Obstetrics & Gynecology Department* |  |
| - Obstetricians/Gynecologists |  |
| - Midwives |  |
| - Nurses |  |
| - Health and social care workers |  |
| Subtotal |  |
| *Pediatric Department/Nursery* |  |
| - Pediatrician |  |
| - Nurses |  |
| - Health and social care workers |  |
| Subtotal |  |
| *Neonatology Department/Nursery* |  |
| - Neonatologists |  |
| - Nurses |  |
| - Health and social care workers |  |
| Subtotal |  |
| Total (all staff) |  |

| **Part G. Protocols on breastfeeding** | | |
| --- | --- | --- |
|  | Yes | No |
| Does the MH have a check list on breastfeeding topics for antenatal classes |  |  |
| Does the MH have the following protocols? |  |  |
| a) Thermal control of the newborn infant |  |  |
| b) Prevention of in hospital neonatal fall |  |  |
| c) Prevention of sudden unexpected postnatal collapse (SUPC) |  |  |
| d) SSC after vaginal delivery |  |  |
| e) SSC after CS |  |  |
| f) Zero separation and rooming-in 24/24 h |  |  |
| g) Contraindications to breastfeeding |  |  |
| h) Responsive exclusive breastfeeding |  |  |
| i) Helping mothers to breastfeed |  |  |
| j) Prevention and management of pain while breastfeeding |  |  |
| k) Management of early neonatal weight loss |  |  |
| l) Supplementing breast milk with formula |  |  |
| m) Prevention of neonatal hypoglycemia |  |  |
| n) How to support breastfeeding in the jaundiced newborn infant during phototherapy |  |  |
| o) Prevention and management of breast engorgement |  |  |
| p) Expression of breast milk |  |  |
| q) Storage of expressed mother’s milk and human donor milk |  |  |
| r) Prevention and management of lactational mastitis |  |  |
| s) Hospital discharge of the breastfed newborn |  |  |

**Annex 1. Questionnaire on rooming-in submitted to mothers at hospital discharge**

|  | Yes | No |
| --- | --- | --- |
| 1. Did your baby stay in your room all the desired time, even 24/24 hour? |  |  |
| 2. Did your baby stay with you all the previous nights? |  |  |
| 3. If your baby was separated from you, did the separation time exceeded 4 hours? |  |  |
|  | Yes | No |
| If the answered was “yes” to all the 3 above listed questions ,the dyad can be considered to have practiced extensive rooming-in |  |  |
